# Meta-Analysis of Paclitaxel-Based Chemotherapy Combined With Traditional Chinese Medicines for Gastric Cancer Treatment

**DOI:** 10.3389/fphar.2020.00132

**Published:** 2020-02-27

**Authors:** Yicong Li, Xinbing Sui, Zeqi Su, Chunyue Yu, Xiaoguang Shi, Nadia L. Johnson, Fuhao Chu, Yuan Li, Kexin Li, Xia Ding

**Affiliations:** ^1^ Dongzhimen Hospital, Beijing University of Chinese Medicine, Beijing, China; ^2^ School of Traditional Chinese Medicine, Beijing University of Chinese Medicine, Beijing, China; ^3^ Department of Cancer Pharmacology, Holistic Integrative Pharmacy Institutes, College of Medicine, Hangzhou Normal University, Hangzhou, China; ^4^ Beijing Research Institute of Chinese Medicine, Beijing University of Chinese Medicine, Beijing, China; ^5^ Department of Surgery, Dongzhimen Hospital of Beijing University of Chinese Medicine, Beijing, China

**Keywords:** gastric cancer, paclitaxel, traditional Chinese medicine, chemotherapy, meta-analysis

## Abstract

This study aimed to compare the efficacy and safety of traditional Chinese medicines (TCMs) combined with paclitaxel-based chemotherapy and paclitaxel-based chemotherapy alone for gastric cancer treatment. Literature searches (up to September 25, 2019) were performed using the Cochrane Library, EMBASE, PubMed, Chinese Science and Technology Journals (CQVIP), Wanfang, and China Academic Journals (CNKI) databases. Data from 14 randomized controlled trials (RCTs), with 1,109 participants, were included. The results indicated that, compared with paclitaxel-based chemotherapy alone, the combination of TCMs and paclitaxel-based chemotherapy significantly improved the tumor response rate (TRR; RR: 1.39; 95% CI: 1.24–1.57; *p* < 0.001, *I* ^2^ = 12%), increased the quality of life based on the Karnofsky Performance Scale score (RR: 1.53; 95% CI: 1.19–1.96; *p* < 0.001, *I* ^2^ = 0%), and reduced the side effects, such as neutropenia (RR: 0.68; 95% CI: 0.55–0.84; *p* < 0.001, *I* ^2^ = 44%), leukopenia (RR: 0.69; 95% CI: 0.54–0.90; *p* < 0.01, *I* ^2^ = 40%), thrombocytopenia (RR: 0.66; 95% CI: 0.46–0.96; *p* < 0.05, *I* ^2^ = 32%), and nausea and vomiting (RR: 0.50; 95% CI: 0.32–0.80; *p* < 0.01, *I* ^2^ = 85%). Hepatic dysfunction (RR: 0.63; 95% CI: 0.33–1.20; *p *= 0.16, *I* ^2^ = 0%), neurotoxicity (RR: 0.64; 95% CI: 0.26–1.55; *p* = 0.32, *I* ^2^ = 0%), and anemia (RR: 0.65; 95% CI: 0.40–1.04; *p* = 0.07, *I* ^2^ = 0%) were similar between the two groups. Evidence from the meta-analysis suggested that compared with paclitaxel-based chemotherapy alone, the combination of TCMs and paclitaxel-based chemotherapy may increase the TRR, improve quality of life, and reduce multiple chemotherapy-related side effects in gastric cancer patients. Additional rigorously designed large RCTs are required to confirm the efficacy and safety of this treatment.

## Introduction

Gastric cancer (GC) is the fifth-most commonly diagnosed cancer and the third leading cause of cancer-related death in the world. In 2018, there were more than 1,000,000 new GC cases and GC resulted in an estimated 783,000 deaths (Bray et al., 2018). Despite the progress in diagnosis and treatment, the initial detection of most GC cases occurs at advanced stages, which leads to poor prognosis, with a median overall survival of 11 months (Wagner et al., 2017). Currently, chemotherapy is widely used as the main treatment for GC. However, the side effects and the development of resistance to chemotherapy in clinical practice reveal its limitations and have prompted more attention to be paid to the study of complementary treatments (Biagioni et al., 2019).

Paclitaxel is a widely used second-line chemotherapy drug for advanced GC (Bang et al., 2017). However, it has a low response rate (16–22%) (Bang et al., 2002) and significant side effects (such as neutropenia and gastrointestinal adverse reactions) (Shitara et al., 2010), so a paclitaxel-based combination regimen may be more beneficial.

Traditional Chinese medicines (TCMs), which can be used as complementary treatments for cancer patients, have been widely used in China for years (Wong et al., 2001; Chen et al., 2016). TCM combinations have been reported to alleviate chemotherapy drug resistance and enhance the efficacy of chemotherapy. However, it remains uncertain whether paclitaxel-based chemotherapy combined with TCMs is more effective than paclitaxel-based chemotherapy alone for GC.

In this study, we aimed to use a meta-analysis to summarize and analyze high-quality RCTs in order to evaluate the efficacy and safety of combination therapy using paclitaxel and TCMs compared to paclitaxel alone for GC. We further aimed to identify the most frequently used TCM herbal compounds in order to provide a reference for the selection of a reasonable TCM regimen for the treatment of GC.

## Materials and Methods

### Study Selection

Databases, comprising PubMed, EMBASE, Cochrane Library, Wanfang, Chinese Science and Technology Journals (CQVIP), and China Academic Journals (CNKI), were independently searched from their inceptions to September 25, 2019 by two reviewers (Yicong Li and Chunyue Yu). The search terms (in English and Chinese) involved the following: “paclitaxel OR Taxol” AND “Chinese herb OR traditional medicine” AND “stomach neoplasm OR gastric neoplasm OR stomach cancer OR gastric cancer”. There was no restriction on the language. The Jadad scale was used to assess study quality (Jadad et al., 1996). 

### Inclusion and Exclusion Criteria

Studies were included if they met the following PICOS criteria: (1) participants: GC patients (diagnosed based on pathology results); (2) intervention: paclitaxel-based chemotherapy regimen combined with TCM; (3) comparator: paclitaxel-based chemotherapy regimen alone; (4) outcomes: tumor response rate (TRR), Karnofsky Performance Scale (KPS) score, and/or side effects (at least one of these outcomes); (5) study design: randomized controlled trial (RCT).

The exclusion criteria were as follows: (1) outcomes not reported clearly or appropriate data could not be extracted; (2) Jadad score <2; (3) duplicate studies by the same authors.

### Data Extraction

All the included studies were screened independently by two reviewers (Yicong Li and Chunyue Yu) to extract the following data: first author (year), study period, sample sizes, tumor, node, metastasis (TNM) stage, TCM intervention, paclitaxel regimen, drug administration route, treatment duration, and outcomes. Any differences were resolved by discussion between the two reviewers, and differences that could not be resolved were settled by a third reviewer (Xinbing Sui).

### Risk of Bias Assessment

We used the Cochrane risk of bias tool (Higgins et al., 2011) to assess the risk of bias of the RCTs. The domains of this tool include selection bias, performance bias, detection bias, attrition bias, and reporting bias. Low, high, and unclear risk of bias indicate that the study met the criteria, did not meet the criteria, and did not provide enough information to make a judgment, respectively.

### Primary Outcomes

Tumor response rate (TRR), containing the criteria of complete response (CR), partial response (PR), stable disease (SD), and progressive disease (PD), was the primary outcome. CR plus PR was also included as TRR. Subgroup analyses were then used to assess whether there was a difference in TRR between the paclitaxel+TCM and control groups in various subgroups of studies, according to administration route (studies were categorized into an oral administration subgroup in which TCMs were taken orally as a decoction or capsules [seven studies] or an injection subgroup [six studies]), TNM stage (studies were categorized into a stage IV-only subgroup [two studies] or other stages subgroup [eleven studies]), treatment duration (studies were categorized into ≤4 weeks [four studies], 4–8 weeks [six studies], and >8 weeks [three studies] subgroups), and the three most commonly used TCM combinations (studies were categorized into overlapping subgroups depending on whether they used a combination of Dangshen and Gancao [eight studies], Dangshen, Gancao, Baizhu and Fuling [seven studies], or Dangshen, Gancao, Baizhu, Fuling, Chenpi, Shanyao, Yiyiren, and Sharen [three studies]).

### Secondary Outcomes

KPS score was used to assess quality of life (QOL). Improvement of QOL was defined as a KPS score increase of ≥10 points after treatment. Side effects, which included blood abnormalities (neutropenia, leukopenia, anemia, and thrombocytopenia), nausea and vomiting, hepatic dysfunction, and neurotoxicity, were also evaluated as secondary outcomes.

### Data Analysis

Cochrane Review Manager (RevMan) software version 5.3 was utilized for statistical analysis. Risk ratio (RR) with a 95% confidence interval (CI) for the dichotomous outcomes was used to estimate the pooled effects. Heterogeneity was estimated by Cochran's Q test and assessed using *I* ^2^. A fixed-effects model was used to estimate the pooled effect when heterogeneity was absent (*I* ^2^ < 50%). Otherwise, a random-effects model was used. *p* < 0.05 was considered significant. A funnel plot was used to assess publication bias regarding TRR data.

## Results

### Literature Search

**Figure 1:**
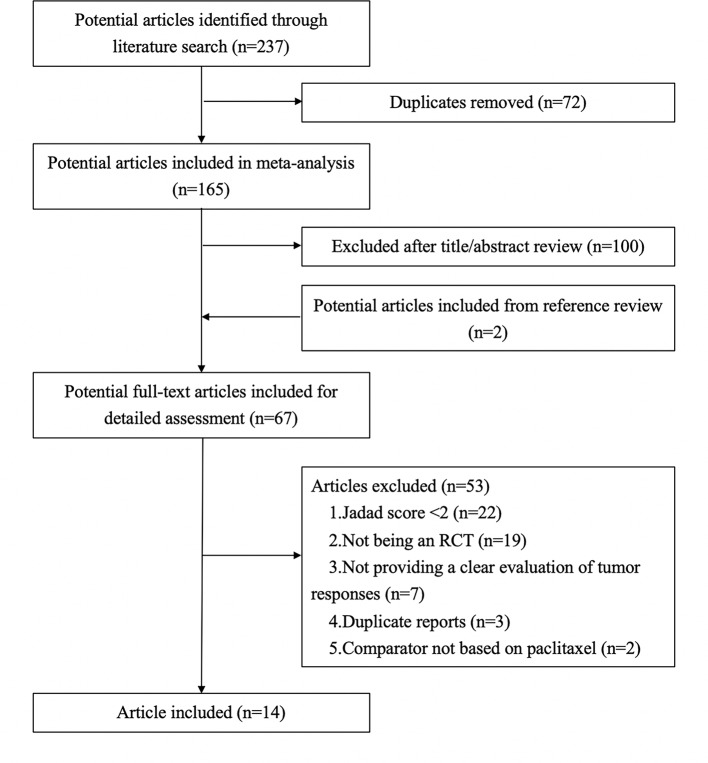
Flow diagram showing study selection for meta-analysis.

As shown in **Figure 1**, 237 articles were retrieved in the literature search. After removal of duplicate articles, 165 articles remained. After reviewing the titles and abstracts, 100 irrelevant articles were excluded. After adding two articles based on a review of the references of the remaining 65 articles, there were 67 articles. The full-text articles were then evaluated based on the inclusion and exclusion criteria, and 53 articles were excluded due to: Jadad score <2 (n = 22), not being an RCT (n = 19), not providing a clear evaluation of tumor responses (n = 7), duplicate reports (n = 3), and comparator not based on paclitaxel (n = 2). Further details are shown in **Supplementary Table 1**. Fourteen articles were finally included in our meta-analysis.

### Study Characteristics

The main characteristics of the 14 included studies are shown in **Table 1**. The publication years ranged from 2009 to 2018 and there were 1,109 included patients, with 502 in the paclitaxel+TCM group and 506 in the control group.

**Table 1:** Characteristics of the included studies.

First Author (Year)	Study Period	Sample size (T/C n)	TNM Stage	Drug Delivery	TCM Duration	TCM Intervention	Paclitaxel Regimen	TRR (T/C n)
Chen (2009)	2005–2007	34/33	IIIb: 23/24; IV: 11/9	Injection	6w	Huachansu injection	TPF: PTX+PDD+5-FU	15/12
Ge et al. (2014)	NR	20/20	Advanced stage	Orally	8w	Jianpixiaozheng decoction	TS: PTX+S-1	10/9
Huang et al. (2018)	2015–2016	50/50	Advanced stage	Injection	18w	Kanglaite injection + Jianpiyiqi decoction	TP: PTX+PDD	35/20
Lai et al. (2018)	2015–2018	30/30	IV	Orally	6w	Shenlingbaizhu decoction	TCF: PTX+CF+5-FU	NR
Li N. J., et al (2016)	2013–2014	25/25	Advanced stage	Orally	4w	Rg3+Shenyi capsule	TCF: PTX+CF+5-FU	17/11
Li et al. (2010)	2007–2009	42/40	Advanced stage	Orally	22d	Liujunzi decoction	TCF: PTX+CF+5-FU	25/23
Li et al. (2016a)	2012–2015	33/32	IV	Injection	8w	Fufangkushen injection + Yiqiyangwei decoction	PTX	27/18
Li et al. (2016b)	2014–2015	50/50	Advanced stage	Orally	8w	Shenlingbaizhu decoction	TS: PTX+S-1	27/22
Liao (2018)	2015–2016	31/31	Advanced stage	Injection	4w	Kang'ai injection	PTX+CF	27/19
Liu and Zhang (2009)	2006–2008	30/30	IIIb: 17/14; IV: 13/16	Injection	20d	Aidi injection	TPF: PTX+PDD+5-FU	16/16
Meng (2019)	2015–2016	50/50	III: 34/36; IV: 16/14	Injection	12w	Fufangkushen injection	PTX	35/15
Tan et al. (2013)	2007–2011	40/46	IV	Orally	6w	Fufangbanmao capsule	PTX+5-FU+LV	19/12
Xue and Mao (2017)	2013–2015	45/45	Advanced stage	Orally	8w	Rg3	TCF: PTX+CF+5-FU	35/28
You and Huang (2009)	2007–2008	22/24	III:6/5; IV:16/19	Orally	24w	Fuzhenghewei liquid medicament	TP: PTX+PDD/TCF: PTX+CF+5-FU	8/8

T, treatment group; C, control group; TNM, cancer staging system; NR, not reported; TCM, traditional Chinese medicine, TRR, tumor response rate; w, week; d, day; PTX, paclitaxel; PDD, cisplatin; 5-FU, 5-fluorouracil; CF, calcium folinate; LV, leucovorin.

### Risk of Bias and Methodological Quality

**Figure 2:**
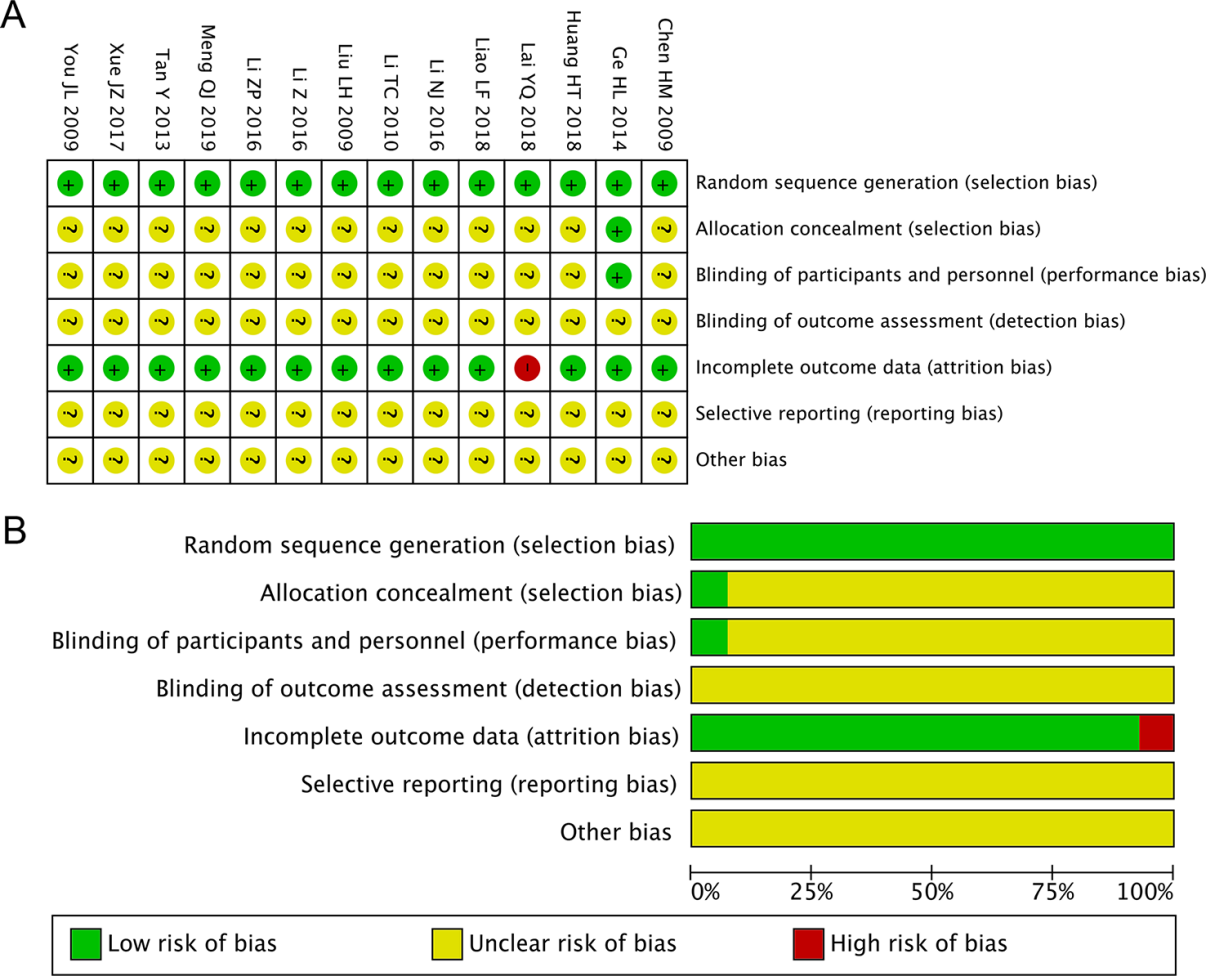
Risk of bias summary and diagram. **(A)** Risk of bias summary: review of authors' judgments about each risk of bias item for all included studies. **(B)** Risk of bias diagram: review of authors' judgments about each risk of bias item presented as percentages across all included studies. Red, green, and yellow indicate high, low, and unclear risk of bias, respectively.

We selected the risk of bias tool provided by the Cochrane Collaboration (Higgins et al., 2011) to assess the risk of bias of the included studies. All of the included studies exhibited bias according to at least one of the bias categories. “Random” or “randomized” or “randomization” was mentioned in all 14 studies, along with descriptions of the specific randomization methods. One study reported allocation concealment and blinding of participants and healthcare providers, but there was unclear blinding of outcome assessment (Ge et al., 2014). One study lacked essential data TRR (Lai et al., 2018), while the other 13 studies reported detailed outcome data. None of the 14 studies provided clear descriptions of detection bias, reporting bias, or other bias. **Figure 2** shows detailed overviews of the scores in each bias category for each study.

### Meta-Analysis of TRR

**Figure 3:**
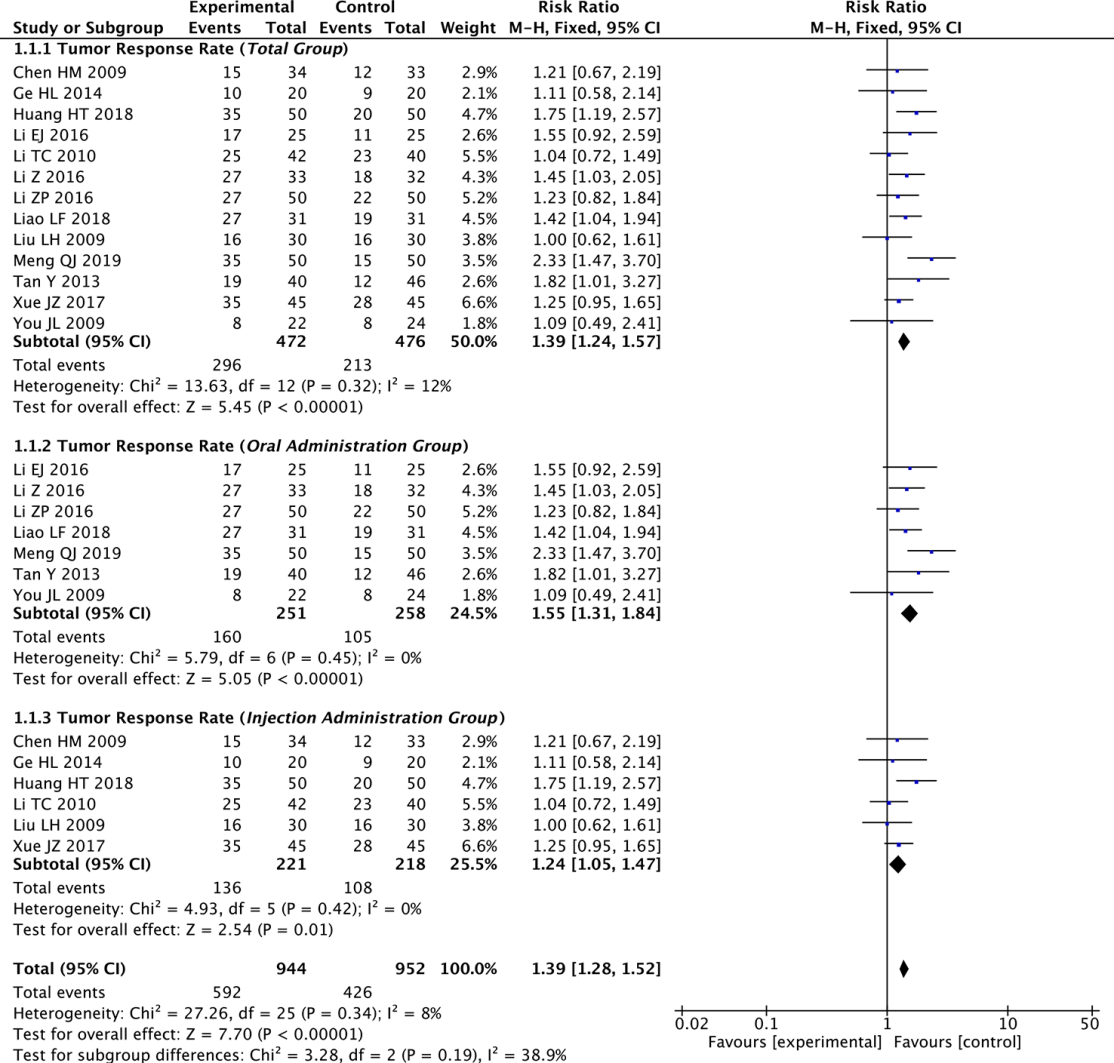
Forest plot of meta-analysis of tumor response rate (TRR) (all studies and subgroups of studies: oral administration subgroup, injection subgroup).

We extracted the TRR data from 13 of the 14 included studies. The fixed-effects meta-analysis showed that the TRR was significantly improved in the paclitaxel+TCM group compared to the control group (RR: 1.39; 95% CI: 1.24–1.57; *p* < 0.001, *I* ^2^ = 12%) (**Figure 3**).

Regarding the administration route subgroup analysis, the TRR was significantly enhanced in the paclitaxel+TCM group compared with the control group in the oral administration subgroup (seven studies; RR: 1.55; 95% CI: 1.31–1.84; *p* < 0.001, *I* ^2^ = 0%) and the injection subgroup (six studies; RR: 1.24; 95% CI: 1.05–1.47; *p* < 0.05, *I* ^2^ = 0%) (**Figure 3**).

**Figure 4:**
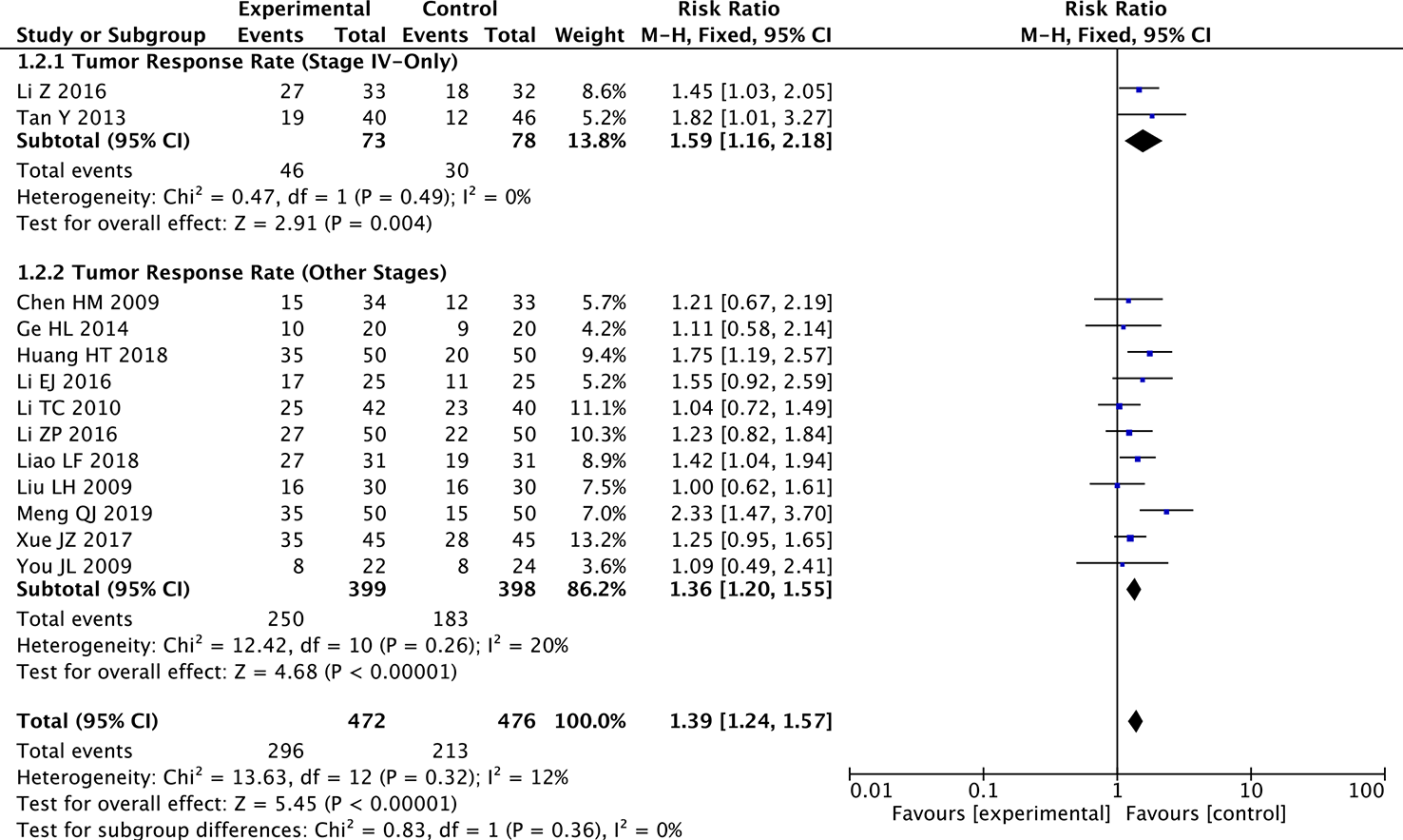
Forest plot of subgroup meta-analysis of TRR according to TNM stages.

Regarding the TNM subgroup analysis, the TRR was significantly improved in the paclitaxel+TCM group compared with the control group in the stage IV-only subgroup (two studies; RR: 1.59; 95% CI: 1.16–2.18; *p* < 0.01, *I* ^2^ = 0%) and the other stages subgroup (eleven studies; RR: 1.36; 95% CI: 1.20–1.55; *p* < 0.001, *I* ^2^ = 20%) (**Figure 4**).

**Figure 5:**
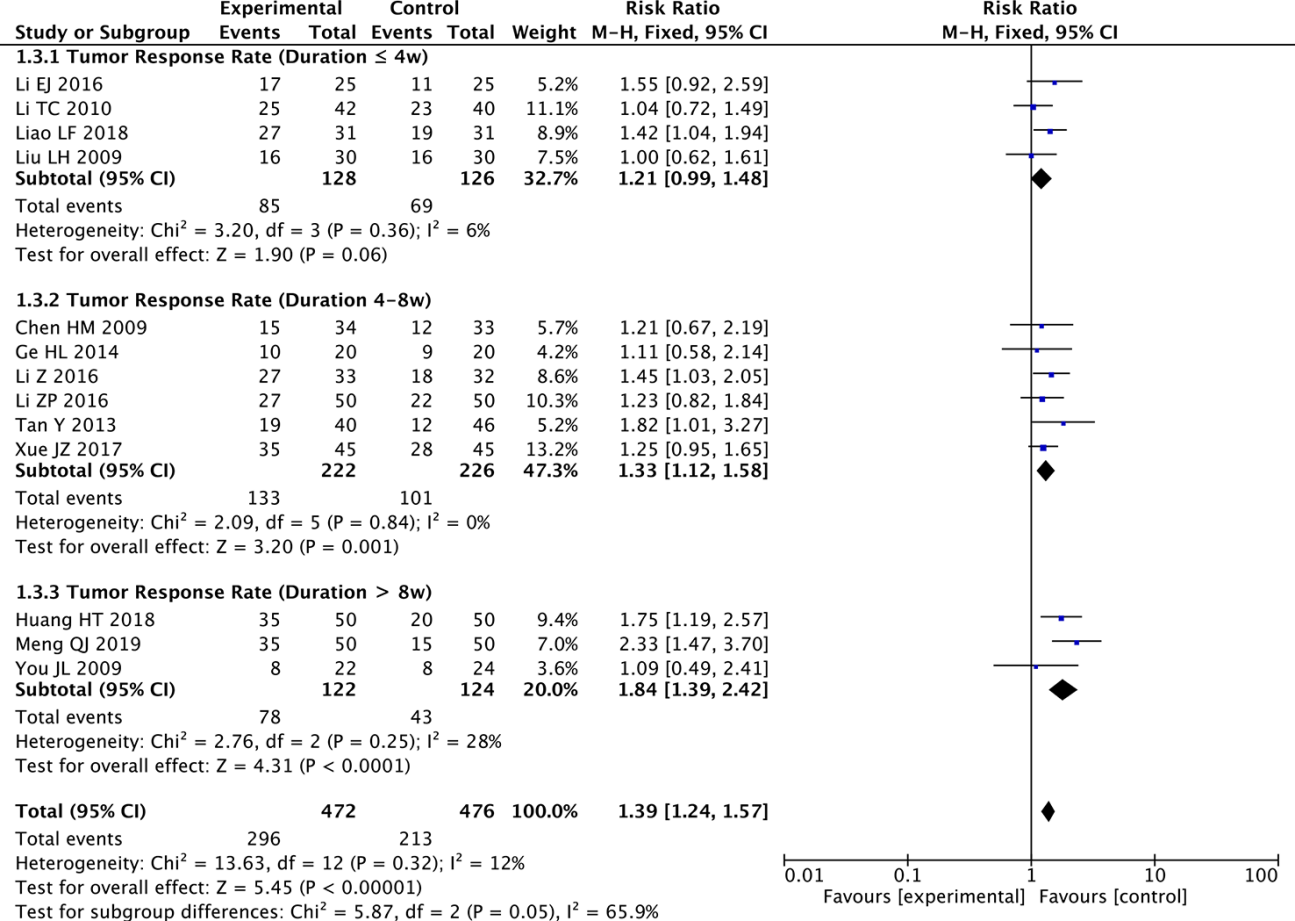
Forest plot of subgroup meta-analysis of TRR according to treatment duration.

Regarding the treatment duration subgroup analysis, there were no significant effects on TRR in the ≤4 weeks subgroup (four studies; RR: 1.21; 95% CI: 0.99–1.48; *p* = 0.06, *I* ^2^ = 6%), but there was significant improvement in the 4–8 weeks subgroup (six studies; RR: 1.33; 95% CI: 1.12–1.58; *p* < 0.01, *I* ^2^ = 0%) and the >8 weeks subgroup (three studies; RR: 1.84; 95% CI: 1.39–2.42; *p* < 0.001, *I* ^2^ = 28%) (**Figure 5**).

### Meta-Analysis of KPS

**Figure 6:**
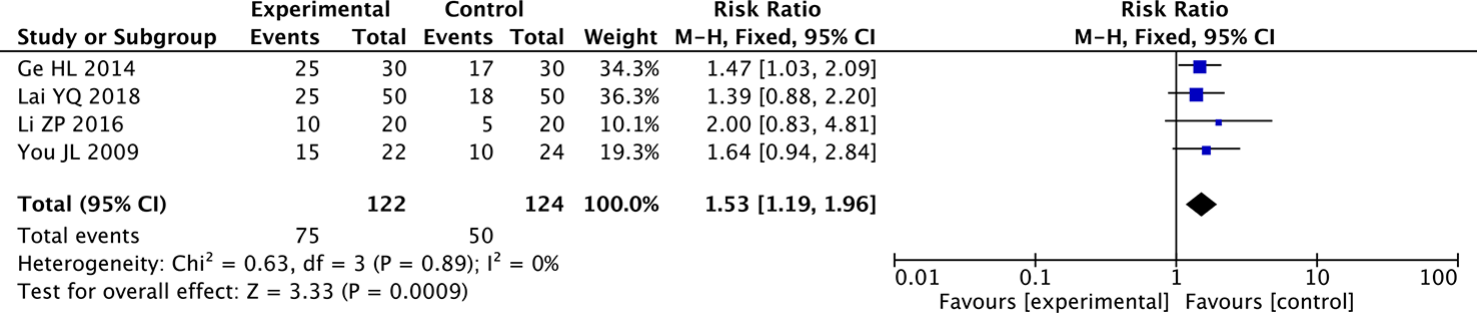
Forest plot of meta-analysis of improvement in Karnofsky Performance Scale (KPS) score (≥10 points).

The fixed-effects meta-analysis showed a significant difference between the two groups in the rate of KPS improvement (≥10 points) (four studies; RR: 1.53; 95% CI: 1.19–1.96; *p* < 0.001, *I* ^2^ = 0%) (**Figure 6**). The KPS was significantly higher in the paclitaxel+TCM group than the control group. The results indicated that, compared with paclitaxel-based chemotherapy alone, combined therapy with TCMs can significantly improve the QOL of patients with GC.

### Meta-Analysis of Blood Abnormalities

**Figure 7:**
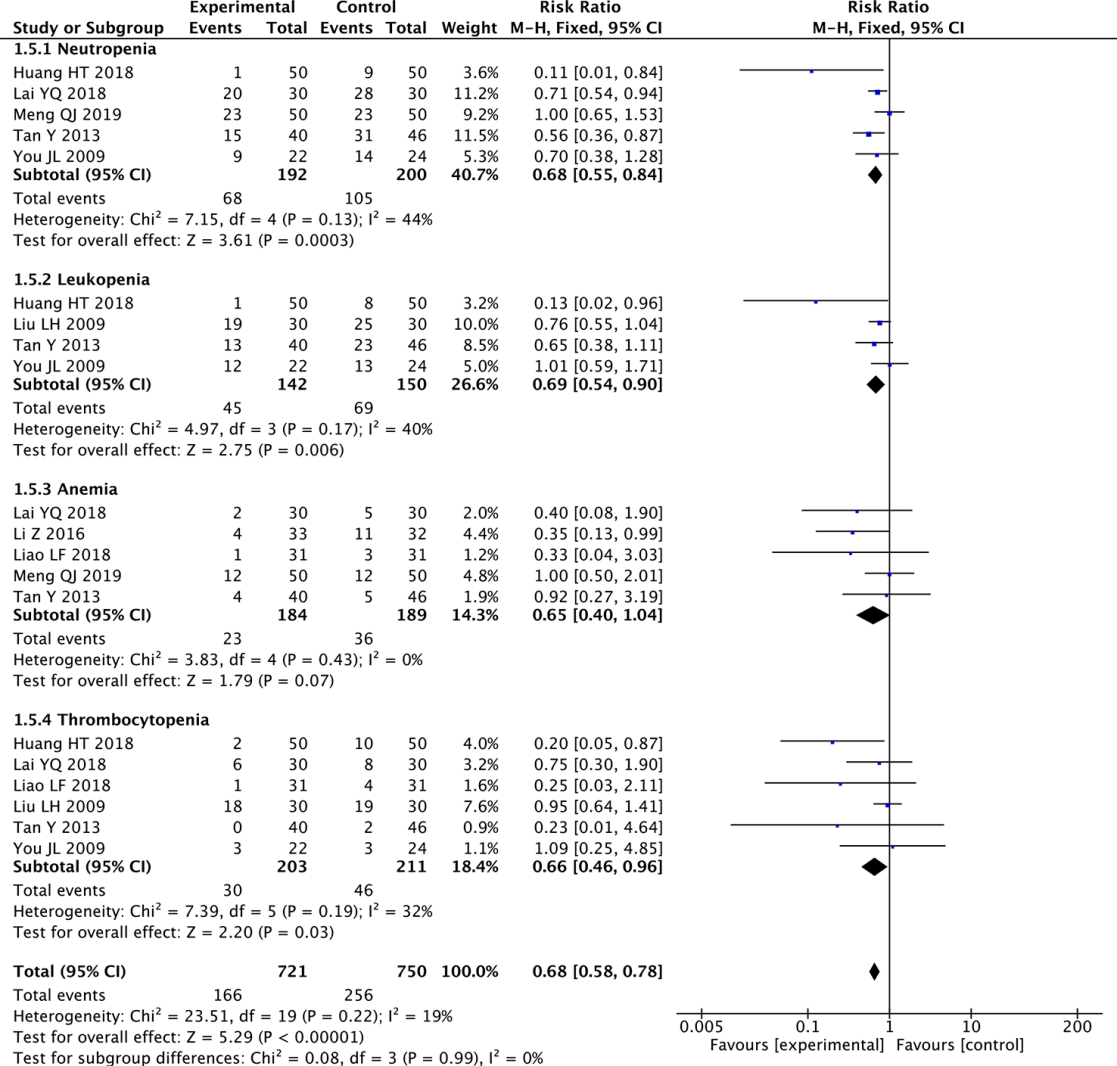
Forest plot of meta-analysis of blood abnormalities.

The fixed-effects meta-analyses showed significant decreases in the paclitaxel+TCM group in the rate of neutropenia (five studies; RR: 0.68; 95% CI: 0.55–0.84; *p* < 0.001, *I* ^2^ = 44%), the rate of leukopenia (four studies; RR: 0.69; 95% CI: 0.54–0.90; *p* < 0.01, *I* ^2^ = 40%), and the rate of thrombocytopenia (six studies; RR: 0.66; 95% CI: 0.46–0.96; *p* < 0.05, *I* ^2^ = 32%) (**Figure 7**). The rate of anemia did not differ significantly (five studies; RR: 0.65; 95% CI: 0.40–1.04; *p *= 0.07, *I* ^2^ = 0%) (**Figure 7**). The results showed that paclitaxel-based chemotherapy combined with TCMs significantly reduced the rate of neutropenia, leukopenia, and thrombocytopenia, but had no significant effect on the rate of anemia during the treatment of GC.

### Meta-Analysis of Nausea and Vomiting, Hepatic Dysfunction, and Neurotoxicity

**Figure 8:**
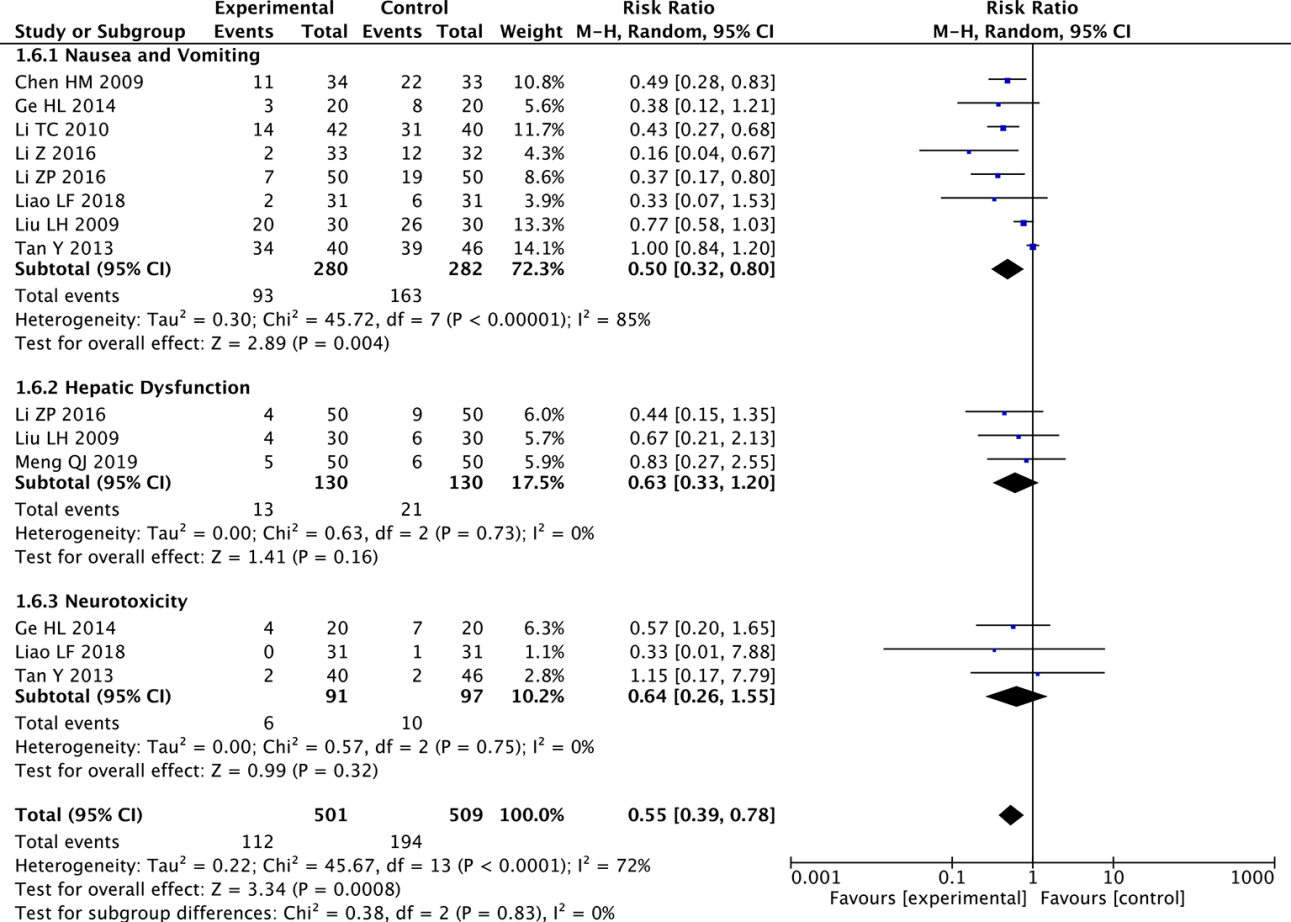
Forest plot of meta-analysis of nausea and vomiting, hepatic dysfunction, and neurotoxicity.

The random-effects meta-analysis showed a significantly lower rate of nausea and vomiting in the paclitaxel+TCM group compared to the control group (eight studies; RR: 0.50; 95% CI: 0.32–0.80; *p* < 0.01, *I* ^2^ = 85%). However, there were no significant differences in hepatic dysfunction (three studies; RR: 0.63; 95% CI: 0.33–1.20; *p* = 0.16, *I* ^2^ = 0%) or neurotoxicity (three studies; RR: 0.64; 95% CI: 0.26–1.55; *p* = 0.32, *I* ^2^ = 0%) (**Figure 8**). Regarding the analysis of nausea and vomiting, a random-effects model was used to calculate the pooled RR (and 95% CI) due to significant heterogeneity (*p* < 0.001, *I* ^2^ = 72%). These results indicated that paclitaxel combined with TCMs can significantly reduce nausea and vomiting compared to paclitaxel-based chemotherapy alone, without causing additional hepatic dysfunction or neurotoxicity.

### TCM Formulae and Frequently Used Herbal Compounds

**Figure 9:**
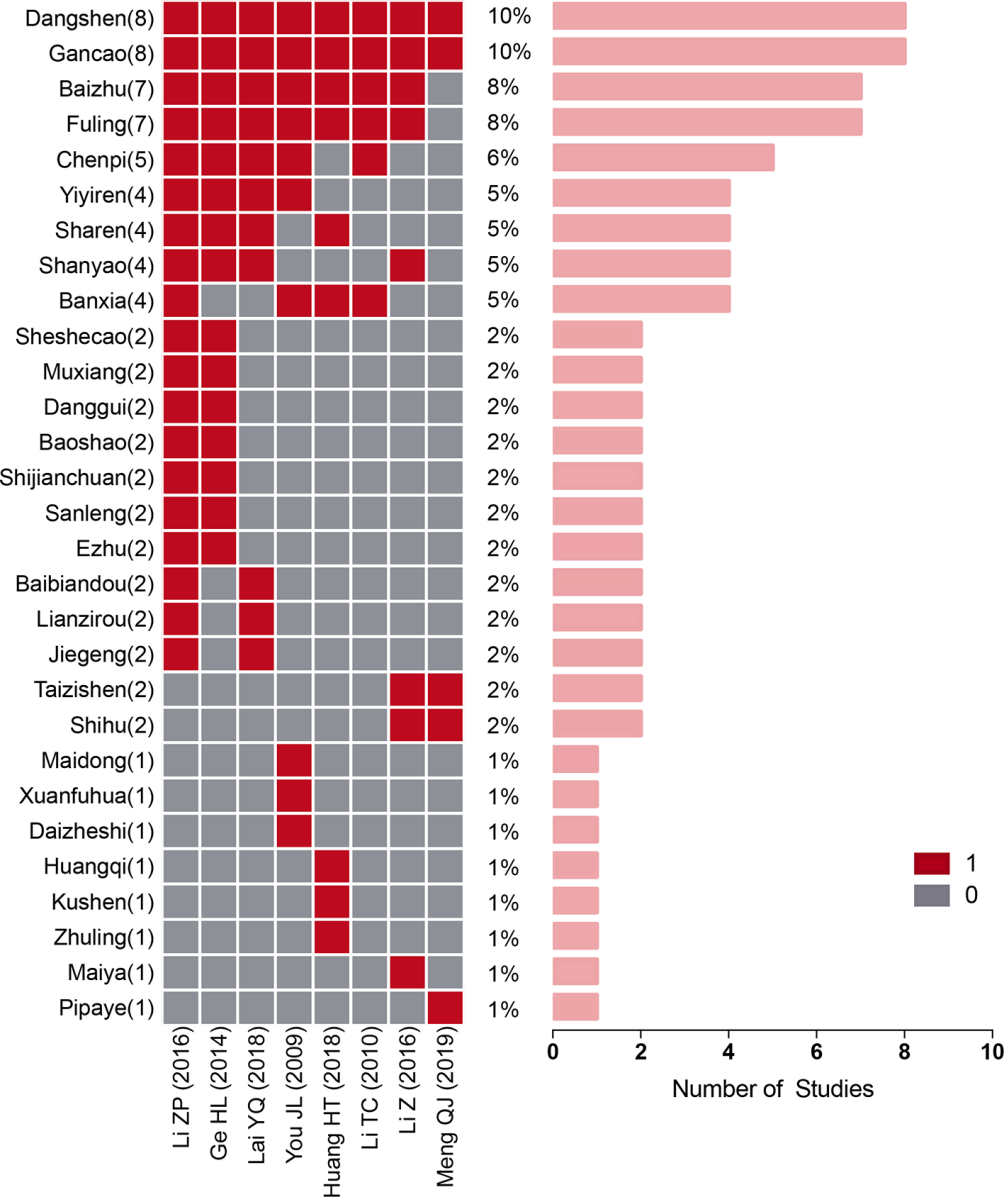
Traditional Chinese medicine (TCM) herbal compounds used in each study.

Among the studies in the oral administration subgroup that used multi-ingredient TCM regimens, there were eight studies with a total of 29 TCM ingredients. Nine of these ingredients were used in four or more formulations. Ordered according to their frequency of use, the TCMs were as follows: Dangshen (n = 8), Gancao (n = 8), Baizhu (n = 7), Fuling (n = 7), Chenpi (n = 5), Banxia (n = 4), Shanyao (n = 4), Yiyiren (n = 4), and Sharen (n = 4) (**Table 2** and **Figure 9**). Using fixed-effects models, we performed a subgroup analysis of the three most commonly used combinations. Regarding the subgroup involving the combination of Dangshen and Gancao, TRR was significantly improved in the paclitaxel+TCM group compared to the control group (eight studies; RR: 1.44; 95% CI: 1.22–1.70; *p* < 0.001, *I* ^2^ = 40%). Moreover, regarding the subgroup involving the combination of Dangshen, Gancao, Baizhu and Fuling, there was a significant improvement (seven studies; RR: 1.31; 95% CI: 1.09–1.56; *p* < 0.01, *I* ^2^ = 0%). Furthermore, regarding the subgroup involving the combination of Dangshen, Gancao, Baizhu, Fuling, Chenpi, Shanyao, Yiyiren, and Sharen, there was also a significant improvement (three studies; RR: 1.30; 95% CI: 1.20–1.41; *p* < 0.001, *I* ^2^ = 0%).

**Table 2:** Name of High Frequency TCMs.

Chinese Name	Pharmaceutical name	Family	No. of Studies
Dangshen	*Codonopsis Radix*	*Campanulaceae*	8
Gancao	*Glycyrrhizae Radix et Rhizoma*	*Leguminosae*	8
Baizhu	*Atractylodis Macrocephalae Rhizoma*	*Asteraceae*	7
Fuling	*Poria*	*Polyporaceae*	7
Chenpi	*Citri Reticulatae Pericarpium*	*Rutaceae*	5
Shanyao	*Dioscoreae Rhizoma*	*Dioscoreaceae*	4
Yiyiren	*Coicis Semen*	*Gramineae*	4
Sharen	*Amomi Fructus*	*Zingiberaceae*	4
Banxia	*Pineelliae Rhizoma*	*Araceae*	4

### Publication Bias

**Figure 10:**
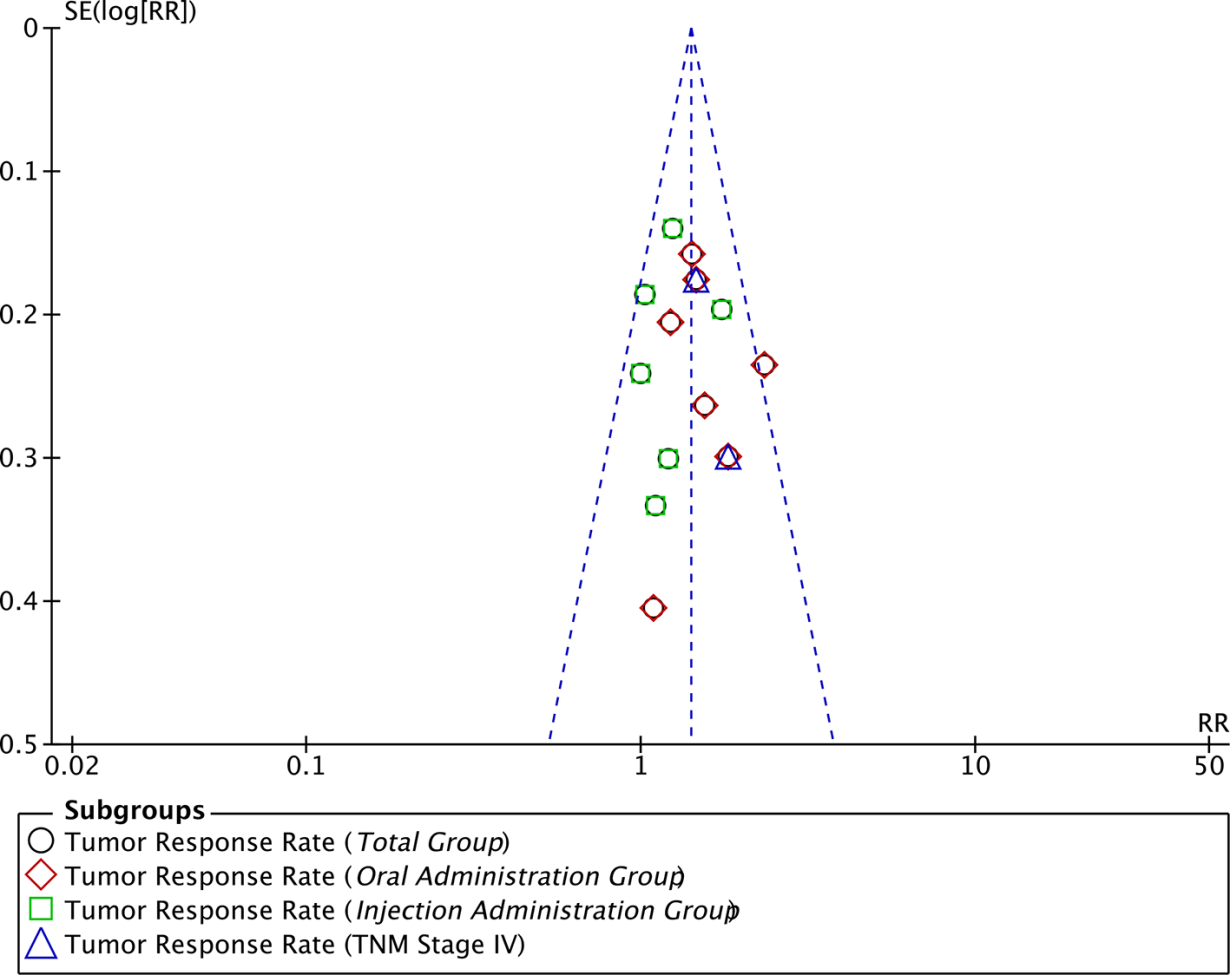
Funnel plot assessing publication bias regarding TRR.

A funnel plot of the 13 studies that reported TRR data was used to assess publication bias (**Figure 10**). The funnel plot was asymmetrical, indicating the existence of publication bias.

## Discussion

Paclitaxel, which is a representative second-line chemotherapy drug for GC, is generally recognized as being able to inhibit cancer (Zhang et al., 2019). However, because of its stronger side effects compared with the first-line chemotherapy drugs, it represents a double-edged sword in GC treatment. This has become an urgent problem to be solved, potentially with TCM combination therapy. Our study showed that, overall, paclitaxel+TCM significantly improved the TRR in GC patients, and it particularly improved the TRR in the subgroups of studies that used oral administration of TCM, included only stage IV patients, and had a long treatment duration. As the primary outcome, TRR can directly reflect the efficacy of chemotherapy regimens on tumors. A meta-analysis by Xie et al. (2013) also showed that TCM (Huachansu) combined with chemotherapy increased the TRR in patients with GC, which is consistent with our findings. In addition, it is necessary to assess QOL to evaluate the effects of chemotherapy combined with TCMs. Among the included studies, four reported the number of patients in each group with a KPS increase ≥10 points. We evaluated the dichotomous variable (KPS improvement) in the meta-analysis, which showed a positive result in the paclitaxel+TCM group compared to the control group.

In addition to enhancing the effect of chemotherapy, combining chemotherapy with TCMs can reduce the side effects and decrease mild drug resistance. Accordingly, our study comprehensively compared the side effects of the two regimens. The blood abnormality results showed that the paclitaxel+TCM group reduced the rates of neutropenia, leukopenia, and thrombocytopenia. Paclitaxel+TCM also alleviated nausea and vomiting after chemotherapy, which shows the positive effect of TCMs on the gastrointestinal system. Furthermore, given that there are a few reports on the adverse effects of TCMs, we evaluated negative effects related to hepatic dysfunction and neurotoxicity. It is gratifying to note that the paclitaxel+TCM group had fewer cases of hepatic dysfunction and neurotoxicity than the groups involving paclitaxel-based chemotherapy alone.

To discern the commonalities between the TCM formulae that were combined with paclitaxel in the various studies, we analyzed the frequency and compatibility of the oral Chinese herbal compounds in the included studies. As the most effective qi tonic among the administered TCMs, the combination of Dangshen and Gancao was used in all eight studies in the oral administration subgroup that used multi-ingredient TCM regimens. In addition, seven of these studies (87.5%) used a combination of Dangshen, Gancao, Baizhu and Fuling, which is the most representative basic qi tonic TCM formula and is known as Sijunzi Decoction. According to these findings, we can infer that when treating GC using paclitaxel-based chemotherapy combined with TCMs, most of the studies invigorated the qi and strengthened the spleen as the standard treatment approach. This is also supported by the existing evidence on TCM treatment for advanced GC and TCMs combined with chemotherapy (Chen et al., 2018). In addition, we found a frequently recurring combination of eight ingredients (Dangshen, Gancao, Baizhu, Fuling, Chenpi, Shanyao, Yiyiren, and Sharen). This combination could be used to guide the prescribing of TCMs in paclitaxel+TCM regimens for the clinical treatment of GC and can be used as a candidate treatment for further RCTs.

An advantage of our study was the use of strict inclusion and exclusion criteria, excluding studies with Jadad scale <2 to improve the quality of the meta-analysis. Furthermore, we not only systematically searched the databases (from their inceptions) for studies in English, but we also searched the databases in Chinese; therefore, the included literature was not limited to English. Additionally, we comprehensively evaluated and compared the side effects of paclitaxel+TCM with paclitaxel-based chemotherapy alone. The results indicate that paclitaxel+TCM is more effective and safer. Finally, we discovered a frequently used combination of herbal compounds, which should be assessed in future RCTs.

Our research has several limitations. There was a lack of large, multicenter, standardized RCTs, and our included studies were mostly small, which may have led to some bias in the outcomes. We look forward to more high-quality RCTs being published in international journals. Moreover, the included studies lacked assessment of the efficacy of paclitaxel+TCM regimens in paclitaxel-resistant patients. In the future, we plan to focus on drug resistance in order to facilitate a more comprehensive evaluation of the role of TCMs in combination therapies.

## Data Availability Statement

All datasets generated for this study are included in the article/**Supplementary Material**.

## Author Contributions

XD, XSu, and YiL contributed to conception and design. YiL, CY, FC, YuL, and KL contributed to article collection, data analysis and manuscript drafting. XSu, ZS, XSh, and NJ contributed to the revised version. The final submitted version has been confirmed by all authors.

## Funding

Our research was funded by the National Natural Science Foundation of China (Grant No. 81630080, 91129714 and 81874380) and the National Key R&D Program of China (Grant No. 2018YFC1704100 and 2018YFC1704106).

## Conflict of Interest

The authors declare that the research was conducted in the absence of any commercial or financial relationships that could be construed as a potential conflict of interest.
