# Supplementary material for: Meta-Analysis of Paclitaxel-Based Chemotherapy Combined With Traditional Chinese Medicines for Gastric Cancer Treatment
Source: Front Pharmacol. 2020 Feb 27;11:132. doi: 10.3389/fphar.2020.00132 (PMC7056897; doi:10.3389/fphar.2020.00132)
Supplement: Supplementary file 1 [file Table_1.docx]

**Supplementary Table 1.** Characteristics of Excluded Studies

| **Study** | **Reason for Exclusion** |
| --- | --- |
| Yang XF, et al. 2011 | Jadad score <2 |
| Jiang JC, et al. 2019 | Jadad score <2 |
| Wang Q, et al. 2009 | Jadad score <2 |
| Lai YQ, et al. 2010 | Jadad score <2 |
| Zheng XK, et al. 2015 | Jadad score <2 |
| Lin ZW, et al. 2007 | Jadad score <2 |
| Zhao YH, et al. 2014 | Jadad score <2 |
| Hu GR, et al. 2016 | Jadad score <2 |
| Zhang YY, et al. 2017 | Jadad score <2 |
| Han SR, et al. 2009 | Jadad score <2 |
| Lin CL, et al. 2011 | Jadad score <2 |
| Bai XL, et al. 2009 | Jadad score <2 |
| Li DR, et al. 2013 | Jadad score <2 |
| Wen AP, et al. 2013 | Jadad score <2 |
| Deng WY, et al. 2016 | Jadad score <2 |
| Li JH, et al. 2015 | Jadad score <2 |
| Huang XN, et al. 2008 | Jadad score <2 |
| Zhao JG, et al. 2009 | Jadad score <2 |
| Li H, et al. 2014 | Jadad score <2 |
| Deng ZJ, et al. 2011 | Jadad score <2 |
| Wang JY, et al. 2013 | Jadad score <2 |
| Tao H, et al. 2014 | Jadad score <2 |
| Ou C, et al. 2016 | not being an RCT |
| Zhao AG, et al. 2010 | not being an RCT |
| Xu Y, et al. 2013 | not being an RCT |
| Yang J, et al. 2005 | not being an RCT |
| Xi JL, et al. 2014 | not being an RCT |
| Yuan GR, et al. 2008 | not being an RCT |
| Zhao JL, et al. 2017 | not being an RCT |
| Pan XF, et al. 2015 | not being an RCT |
| Zhao JM, et al. 2007 | not being an RCT |
| Kong TD, et al. 2014 | not being an RCT |
| Liu XM, et al. 2008 | not being an RCT |
| He RQ, et al. 2015 | not being an RCT |
| Jin XF, et al. 2016 | not being an RCT |
| Lan SL, et al. 2015 | not being an RCT |
| Liu F, et al. 2014 | not being an RCT |
| Ling SL, et al. 2010 | not being an RCT |
| Fan M, et al. 2008 | not being an RCT |
| Yang LJ, et al. 2009 | not being an RCT |
| Wang RP, et al. 2014 | not being an RCT |
| Wang J, et al. 2016 | not providing a clear evaluation of tumor responses |
| Huang S, et al. 2017 | not providing a clear evaluation of tumor responses |
| Aoyama T, et al. 2014 | not providing a clear evaluation of tumor responses |
| Jia YY, et al. 2014 | not providing a clear evaluation of tumor responses |
| Wu JC, et al. 2015 | not providing a clear evaluation of tumor responses |
| Ma CZ, et al. 2014 | not providing a clear evaluation of tumor responses |
| Chen GN, et al. 2016 | not providing a clear evaluation of tumor responses |
| Ge HL, et al. 2014 | duplicate reports |
| Zhao J, et al. 2017 | duplicate reports |
| Lv XM, et al. 2013 | duplicate reports |
| Ye YC, et al. 2015 | comparator not based on paclitaxel |
| Fu Y, et al. 2015 | comparator not based on paclitaxel |
